# The Influence of Light Hypothenar Contact during a Reaching Movement on the Centre of Pressure (COP) Forward Displacement

**DOI:** 10.1371/journal.pone.0055360

**Published:** 2013-02-05

**Authors:** Darja Rugelj, Jože V. Trontelj, Vojko Strojnik

**Affiliations:** 1 Faculty of Health Sciences, University of Ljubljana, Ljubljana, Slovenia; 2 Institute for Clinical Neurophysiology, University Clinical Center, Ljubljana, Slovenia; 3 Faculty of Sports, University of Ljubljana, Ljubljana, Slovenia; University of South Australia, Australia

## Abstract

The purpose of this study was to evaluate the effect of additional light hand contact (F<1 N) in the region of the hypothenar eminence on forward movement of the center of pressure (COP) and dominant hand. Subjects sled their hypothenar eminence on a vertically-oriented pressure sensitive board while reaching forward beyond their arm length. In the two separate experiments forty nine healthy, college-aged volunteers participated in the study. Thirty subjects (mean age of 22.2±2.4 years, 6 male and 24 female) participated in the experiment on level ground and nineteen subjects (22±2.6 years, 5 male and 14 female) in the experiment on an elevated surface. The forward displacement of the COP was significantly larger (p = 0.002) when subjects were allowed to slide with the hand as compared to no contact when the activity occurred on level ground (84±10 mm and 79±11 mm, respectively), and on a one meter elevated surface (71±17 mm and 65±21 mm, respectively). The maximal forward reach of the dominant hand was significantly greater when subjects were allowed to slide with the hypothenar eminence as compared to the no contact condition on the level ground (336±35 mm and 344±38 mm, respectively, p<0.02), and on the one meter elevated surface (298±58 mm and 307±58 mm, respectively, p<0.01). This data indicate that subjects were able to use additional haptic information from the hypothenar region to bring their COP and dominant hand further forward while standing on level ground as well as on a one m elevated surface.

## Introduction

Control of upright posture and additional voluntary movements requires integration of afferent flow from different sources. The most important sources are visual, vestibular and proprioceptive systems that provide information about the spatial orientation of the body relative to the supporting surface and environment [1]. An important source of information is the skin of the hand that provides so called haptic perception. The influence of haptic cues is well documented. Holden et al. [2] demonstrated that light touch of the index finger with a stable surface, when the applied contact force was too small to provide significant mechanical stabilization, can compensate for the absence of vision and can decrease mean sway amplitude and path length of the center of pressure (COP) in one-legged standing. Also, in the tandem Romberg stance Jeka and Lakner [3] discovered that, when subjects’ finger tips are allowed light contact with a stationary bar, the mean medio-lateral sway amplitude is significantly greater in no contact compared to contact conditions in both vision and no vision conditions. The utility of light finger contact for the regulation of the stability of medio-lateral COP sway was observed also in normal upright posture, when subjects stood with their feet parallel, and results were similar to that of inherently unstable posture such as tandem and one leg stance [4]. Light touch is an aid to stabilization of the COP in healthy, young, able-bodied subjects [2], [3] as well as in different conditions where sensory input is decreased from one or more sensory channels. For instance, in blind subjects [5], in subjects with vestibular loss [6] and in subjects with peripheral neuropathy [7] light touch decreased the sway of COP. The reduction of postural sway while touching a stationary object has been reported to be greater in older compared to younger persons [8]. Even touching one’s own body with the finger tips on the thigh was reported to cause decreased postural sway and decreased average velocity of the COP [9].

Less attention has been devoted to the use of haptic clues when the contact surface is moving or the subjects are reaching beyond their arm length. Touch contact of a walking cane which is not rigidly fixed on the floor decreases postural sway in the medio-lateral direction in sighted individuals with their eyes closed and in blind individuals [5]. Albertsen et al. [10] report that light grip with three fingers on a movable handle (stick) is as effective as when the handle was fixed in decreasing COP movement. Interpersonal light touch is another paradigm in research of the influence of light touch with movable surfaces on postural sway. Interpersonal light touch of the fingertips reduces postural sway in elderly subjects though to a lesser extent than contact with a stationary surface [11]. The location of the interpersonal contact also plays an important role in the reduction of postural sway, namely the reduction of sway is greater with shoulder compared to finger contact [12]. Additionally, the utility of light touch was demonstrated during treadmill walking and resulted in stabilization of the center of mass [13].

Understanding the modifications of postural control on an elevated support surface is important for understanding the postural threat imposed by standing on these elevated surfaces. It is well documented that subjects standing on various heights modify movement of the COP [14], [15], [16]. The amplitudes of COP sway in the antero-posterior and medio-lateral directions decrease linearly as the postural threat increases from lower surfaces (40 cm) to higher surfaces (160 cm) above ground level [14]. The decrease of the sway amplitude is accompanied by increases in the frequency of the oscillations [17], [15] and decrease in the root mean square of the COP [17]. The magnitude of displacement of the center of mass (COM) in the antero-posterior and medio-lateral directions is reduced as postural anxiety increases when surface heights were 60 cm and 160 cm [16]. A strong relationship between anxiety and postural sway was reported when subjects stood on high surfaces. Those who reported higher levels of anxiety had more pronounced postural sway [18].

In contrast, some studies did not find differences in antero-posterior and medio-lateral sway as expressed by RMS between standing on ground level and on a 9 m high surface [19]. And no change was observed in the sway area and path of the COP in standing on 1 m, 2 m and 10.22 m tall surfaces [20] and no significant differences of COP amplitudes and sway frequencies were observed between 1m high and ground positions [21].

The other change of COP position as a result of standing on elevated surfaces is the position of the COP relative to the base of support. The subjects assumed the position that enabled them to position the COP posteriorly compared to the position of the COP when standing on level ground [14], [21]. Compared to the low threat condition the position of the COP was moved significantly backward - further away (1.2 cm) from the edge of the platform [22]. The backward shift of the mean antero-posterior position of the COP (0.76 cm) was obvious in the high threat conditions [17], [15]. Nakahara and his coworkers [20] placed subjects on elevated surfaces of 1 m, 2 m and 10.22 m. They found that at all heights subjects transferred their COP significantly backwards.

Besides standing, some tasks in different occupational situations are performed while reaching and leaning forward are required. Reaching while standing on level as well as on elevated surfaces is usual in construction occupations, house painting, as well as in the home environment where people need to reach objects beyond their arm length and/or above head height where a chair or ladder is therefore needed. The effect of light touch on the center of pressure and hand excursions during reaching beyond arm length has not been studied. The main purpose of our study was to investigate the effect of light touch (slide) of the hypothenar eminence of the dominant hand during a reaching movement in conditions where reaching movement was performed on level ground and on a one meter elevated surface. To address this, we measured COP displacement in antero-posterior direction in two conditions: no touch (control) and light touch. Presumably, when additional afferent information from skin afferents is added subjects can bring their COP further forward. Therefore, we hypothesized that during reaching movements, light hypothener eminence touch on a stable surface would result in increased movement of the COP anteriorly (forward) compared to no touch conditions.

## Methods

### 1. Participants

In the two separate experiments forty nine healthy volunteers, all college students and aged 22±2.5 years participated in the study. Thirty subjects participated in the experiment on the level ground (mean age of 22.2±2.4 years, 6 male and 24 female) and nineteen subjects (22±2.6 years, 5 male and 14 female) in the experiment on the elevated surface. All subjects were right-handed and had no known musculoskeletal or neuromuscular impairments that might affect their ability to maintain balance. The study was approved by the Slovenian Medical Ethics Committee. Informed written consent was obtained from all participants in the study.

### 2. Experimental Procedure

**Figure 1:**
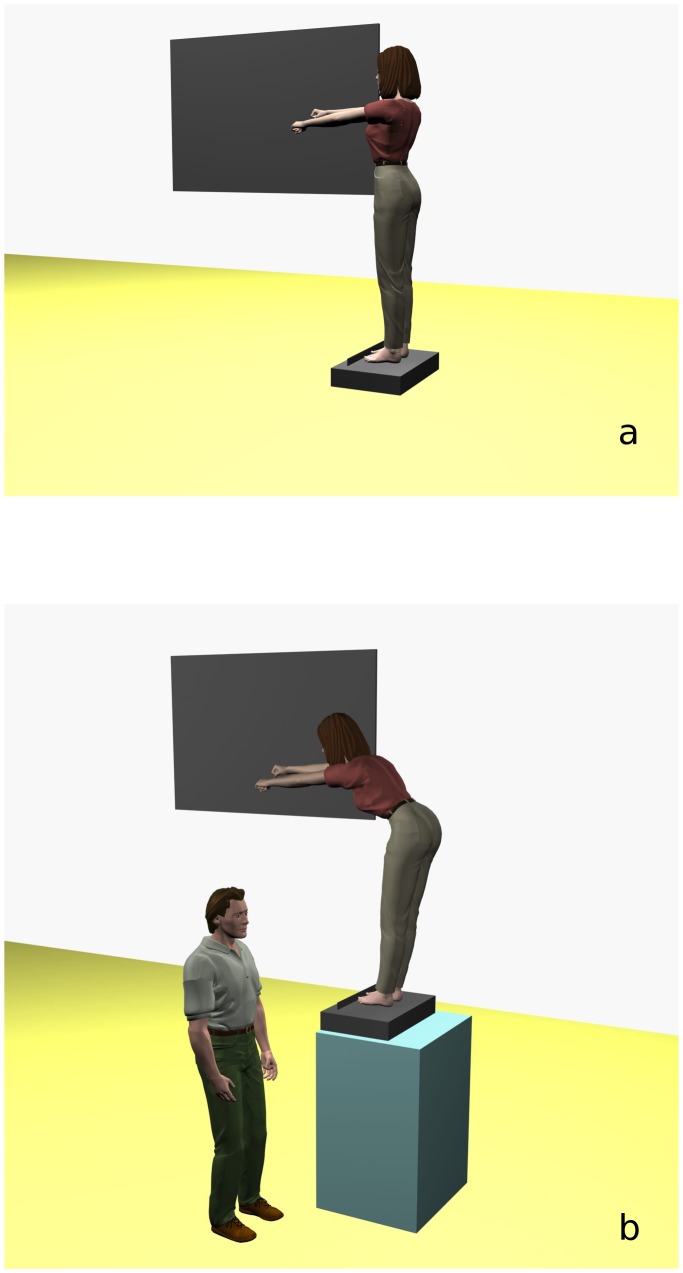
The experimental set up of both experiments. The experimental set up for the reaching forward movement (a) at the level ground and (b) at the height of 1 m. A pressure sensitive board was mounted into the vertical board that was provided for the sliding purpose.

To familiarize with the reaching movement and to exclude learning effects subjects first performed a series of four no contact and four contact reaching movements on the level ground. Thereafter, in both experiments (on level ground and on a one meter elevated surface) subjects performed three sets of four reaching movements - first without sliding with the hypothenar eminence on a vertically-oriented board, second with sliding of the medial part of the hypothenar eminence on the sliding board that was placed at shoulder height (Figure 1) and third they repeated the reaching movement without sliding on the board, which served as a control to exclude possible learning effects in the analysis and interpretation.

A marker (white square 8 mm×8 mm on a black background) was fixed above the head of the third metacarpal of the right hand. Participants were instructed to stand erect on the force platform bare-footed with the right hand oriented towards the sliding board (Figure 1), facing forward with their eyes opened. To ensure that the same foot position could be repeated on each trial a plastic bar was mounted on the platform 7 cm off the border of the force platform. Consistent vertical postural alignment before subjects began the movement was monitored with a 200 cm long wooden plate held perpendicular to the force platform and along the subjects’ back. Both arms were positioned at 90° flexion and hands were closed to a fist. Subject were then instructed to reach forward as much as possible and not to lift their heels off the platform. We used a modified protocol of functional reach [23], [24]. In the contact conditions subjects first touched the vertical board with their hypothenar eminence and then slid with it while reaching forward beyond arm length. After reaching the maximal forward position subjects kept the final position for two seconds and afterwards returned to the erect standing position. The safety of the subjects in the experiment on the elevated surface was ensured by the presence of an able-bodied sportsman standing next to the subject who was ready to prevent falling of the test subjects. Subjects were blind to the function of this “technician”.

### 3. Instrumentation

Displacement of COP in the antero-posterior direction was measured with a Kistler 9286AA (Winthertur, Switzerland) force platform. The platform was connected to a portable computer with DasyLab software (Measurement Computing, Norton, MA, USA) where the data were captured and stored. The force platform was located on a 85 cm tall wooden box (Figure 1).

To ensure sliding with the hypothenar eminence with forces less than 1 N an additional 25×50 cm force plate was mounted into the vertical sliding board (Figure 1). This force plate was constructed for the purpose of this study and was calibrated for a sensitivity between 0.1–5 N. The measured force that exceeded 1 N was signaled by a red led light located on top of the vertical board. If the applied force was greater than 1 N the trial was disregarded and subjects were instructed to repeat the trial.

The excursions of the hand during reaching movements were captured using a 2-dimensional optometric measurements with a digital video recorder Sony super 8 (Sony, Japan). The camera was positioned perpendicular to the motion plane with its axis aligned to the expected end zone of hand marker position. The analysis of the acquired pictures was performed with the Ariel performance analysis system software (APAS, Ariel Dynamics, USA). The other portable computer was connected to the digital video camera. With the use of Adobe Premiere 6.5 software (Adobe Systems Incorporated, San Jose, CA USA) we captured the video directly on a hard disk. With the APAS software we digitized and measured the distances of the right hand.

### 4. Data Processing and Variables

**Figure 2:**
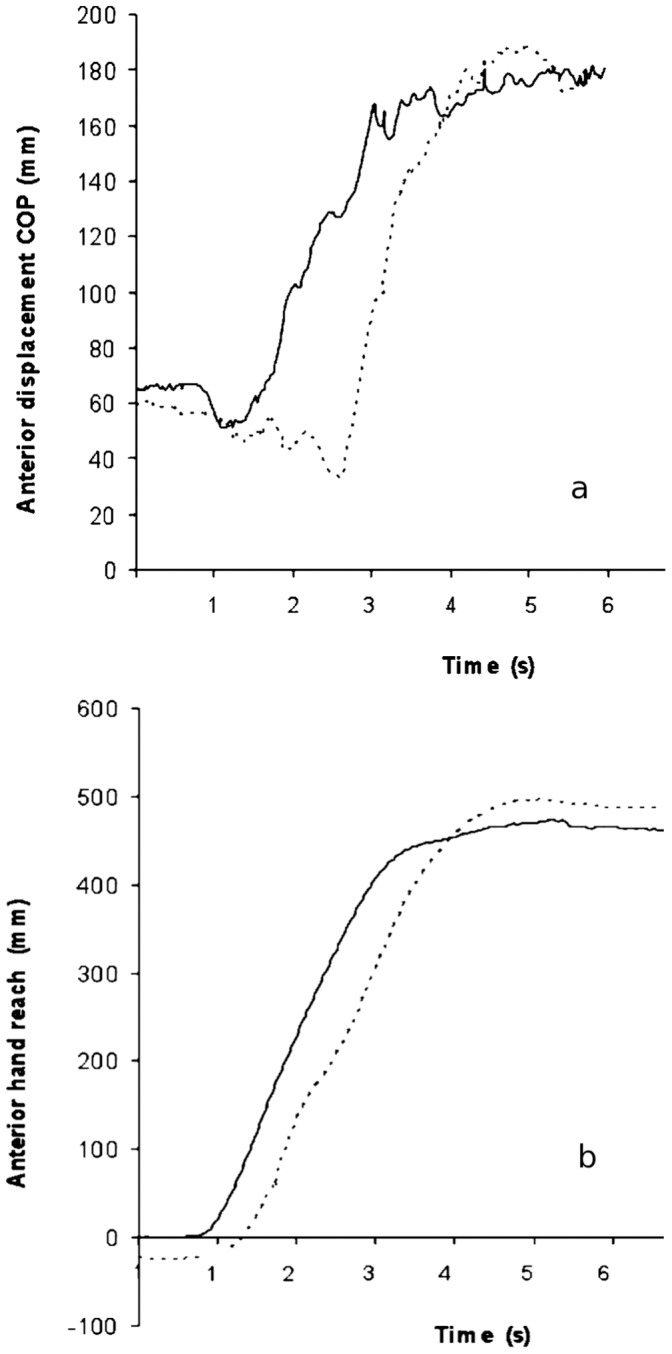
Traces of the anterior displacement of the COP and dominant hand. Traces of the anterior displacement of the COP (a) in no touch (solid line) and light touch conditions and traces of foreword movements of the dominant hand. (b) Trace of hand anterior displacement during reaching movement in no touch (solid line) and light touch conditions (dotted line).

With the specially designed programme in Dasy lab software we marked the points in the trace of the COP and hand movements (Figure 2). The marked events were: initial deflection from the baseline COP position at the beginning of the movement, maximal backward and forward point, and a 2 second point after maximal deflection. All the data were visually inspected for correctness by one of the authors (DR) and accepted or corrected for further analysis. Six variables for COP and hand movements were derived from this data. The analysed variables were: displacement of the COP in the antero-posterior direction from the resting position to: 1) its initial backward movement at the beginning of the reaching movement, 2) its end position that was reached during reaching movement, 3) its average position during 2 second holds of the reach and 4) sway of the COP during hold of the end position expressed as path length. For hand movement the analysed variables were: 1) its end position of the reaching movement and 2) its average position during 2 second holds. In each experimental condition subjects performed 4 consecutive trials. For the analysis the average of these four consecutive trials for each variable was used.

### 5. Statistical Analysis

**T**he position of the COP on the force platform could not be standardised for all of the participants due to different foot/sole sizes of the subjects. Only the anterior position of the big toes was fixed between trials and between subjects, therefore only pair-wise comparisons (within subjects) could be obtained. The nature of the variables, i.e. the amount of COP and hand displacement for each subject in each condition, required within subject comparisons, therefore we performed two sets of paired sample t - tests. First the touch and no touch conditions and second the both no touch conditions were compared. The latter served as a control to exclude potential learning effects since the order of the trials was not randomised. The significance level was set to alpha = 0.02 to allow for Bonferoni adjustments for repeated tests. For the statistical analysis of this data we used SPSS.17 (SPSS Inc., Chicago, IL ZDA) and Microsoft Excel 2003 (Microsoft Inc, Redmond; WA, ZDA).

## Results

In the results section besides the two main variables (maximum and average COP displacement (Fig. 2) additional four COP and two hand movement variables are presented to describe how reaching movements were achived. Results of both experiments are divided in three sections: motion of the COP in the antero-posterior direction, motion of the hand in the antero-posterior direction and repeated no touch measurements. A typical recording of the movements of COP and hand during performance of reaching movement is shown in Fig. 2.

### 1. Experiment one - on the Level Ground

#### Forward movements of the COP

**Figure 3:**
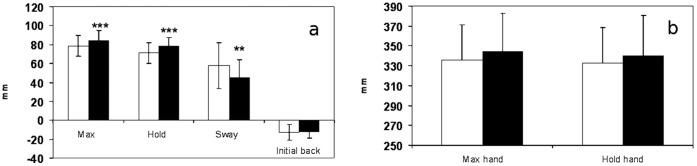
Mean forward displacement of the COP and dominant hand on level ground. (a) Mean forward movement of the COP on a level ground in no touch (white) and touch (black) conditions for variables: Max - forward displacement of the CoP, Hold – average position of the COP in the end position that lasted 2 seconds; Sway - of the COP during a 2 second hold of the reach expressed as path length, Initial back - initial backward displacement of the COP during reach).(b) Mean movement of the hand during the reaching movement in the no touch (white) and touch (black) conditions. The significant differences are marked as **p<0.01; ***p<0.001.

The movement of the COP associated with reaching forward with both hands typically began with backward displacement. It ranged in average from 12.6±8.6 mm in the no touch condition and 11.8±6.8 mm in the touch condition. The difference between the two was not statistically significant (p = 0.628). Maximal forward displacement of the COP, expressed as the maximal end position of the COP, is an average 5.7±2 mm more anterior in the light touch condition as compared to the no touch conditions (Figure 3a), the difference was statistically significant (p<0.001).

During the holding of the end position for 2 seconds the COP moved posteriorly an average of 7.2±2 mm in the no touch conditions, while in the touch condition it moved backwards by 6.4±1.7 mm. The end position of the COP during holding of the reaching movement was significantly more anterior in the touch as compared to no touch conditions (p<0.001), 77.9±9.6 mm and 71.4±10.9 mm, respectively. During this holding of the end position COP swayed and was expressed as the path length of the COP. In the no touch condition sway (59±24.1 mm) was significantly longer (p = 0.003) as compared to the touch (44±19.2 mm) condition (Figure 3).

#### Reaching movement with the hand

The maximal forward reach of the hand with light touch was slightly more anterior (p = 0.024) as compared to no touch reach with an average difference of 8±7.1 mm (Figure 3b). During 2 seconds of holding the end position the hand moves an average of 4±3 mm backwards in the no touch condition and 4±2 mm in the touch condition. The average position during holding was slightly more anterior with touch but did not reach significance (p = 0.085) as compared to no touch, 340.4±40.5 mm and 332.7±35.5 mm, respectively (Figure 3b).

#### Repeated no touch movement

To exclude possible learning effects and to overcome the lack of randomisation of the movement order we performed the third set of measurements with the no touch condition (repetition of the first set of measurements). The time for COP movements, end and average position of the COP and end and average position of the hand, did not differ between the two conditions (Table 1). The results showed that there were no differences between the two no touch conditions.

**Table 1:** The amount of the COP and hand foreword displacement during reaching movement for first and second no touch conditions.

	1st notouch		2nd notouch		P-value	
**Level ground**						
End position of the COP (mm)		78.6±10.9		80±13.1		0.534
Average position of the COP (mm)		71.4±10.9		72.7±12.9		0.502
End position of the hand (mm)		336±35.3		335.4±39		0.810
Average position of the hand (mm)		332.7±35.5		329.5±42.4		0.385
**One meter elevated surface**						
End position of the COP (mm)	64.7±21.2		64.5±21.3		0.877	
Average position of the COP (mm)	56.9±21.1		56.4±20.9		0.654	
End position of the hand (mm)	298.3±58.3		294.1±55.4		0.110	
Average position of the hand (mm)	294.3±57.9		289.96±55.5		0.113	

P-value for the comparison of the 2 no touch conditions is given for the two experiments (level ground and one meter elevated surface).

### 2. Experiment Two - on the One Meter Elevated Surface

#### Forward movements of the COP

**Figure 4:**
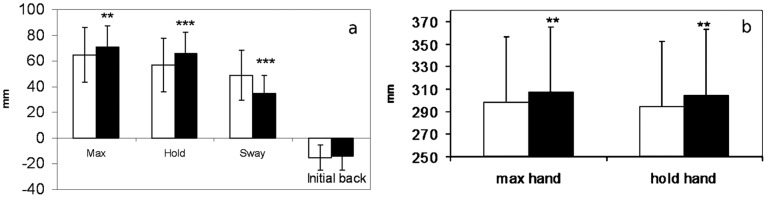
Mean forward displacement of the COP and dominant hand on elevated surface. (a) Mean forward movement of the COP on a 1 m elevated surface in no touch (white) and touch (black) conditions for variables: Max - forward displacement of the CoP, Hold – average position of the COP in the end position that lasted 2 seconds; Sway - of the COP during a 2 second hold of the reach expressed as path length, Initial back - initial backward displacement of the COP during reach).(b) Mean movement of the hand during the reaching movement in the no touch (white) and touch (black) conditions. The significant differences are marked as **p<0.01; ***p<0.001.

The movement of the COP associated with reaching forward with both hands typically began with backward displacement. It ranged in average from 15±10 mm in the no touch condition and 14±11 mm in the touch condition. The difference between the two was not statistically significant (p = 0.22). Maximal forward displacement of the COP, expressed as the maximal end position of the COP, is an average 6±7 mm more anterior in the light touch condition as compared to the no touch conditions (Figure 4a), the difference was statistically significant (p = 0.002).

During the holding of the end position for 2 seconds the COP moved posteriorly an average of 8±2 mm in the no touch conditions, while in the touch condition it moved backwards by only 5±3 mm. The end position of the COP during holding of the reaching movement was significantly more anterior in the touch as compared to no touch conditions (p<0.001), 66±16 mm and 57±21 mm, respectively. During this holding of the end position COP swayed and was expressed as the path length of the COP. In the no touch condition sway (49±19 mm) was significantly longer (p<0.001) as compared to the touch (35±14 mm) condition (Figure 4).

#### Reaching movement with the hand

The maximal forward reach of the hand on the 1 m elevated surface with touch was more anterior (p<0.01) as compared to no touch reach with an average difference of 9±14 mm (Figure 4b). During 3 seconds of holding the end position hand moves an average of 4±3 mm backwards in the no touch condition and 3±2 mm in the touch condition. The average position during holding was significantly more anterior with touch (p<0.004) as compared to no touch, 307±58 mm and 298±58 mm, respectively (Figure 4b).

#### Repeated no touch movement

To exclude the learning effect and to overcome the lack of randomisation of the movement order we performed the third set of measurements with the no touch condition (repetition of the first set of the measurements). The time for COP movements, end and average position of the COP and end and average position of the hand did not differ between the two conditions (Table 1). The results showed that there were no differences between the two no touch conditions.

### 3. Between Experiments Comparisons

The differences of the maximal end positions of the COP during reaching movements were calculated for both experiments. The average increase of the COP forward displacement was 5.7±7.8 mm on the ground and 6.1 mm±7.3 mm on the elevated surface. When the results of the two experiments are normalised and are gains expressed as percentage increases, results indicate gaiter gain on the elevated surface as compared to level ground experiment (an average 13±34 per cent and 8±10 per cent respectively).

## Discussion

The purpose of this study was to evaluate whether light contact of the hypothenar eminence of the dominant hand during reaching movement allowed subjects to bring their COP and the hand further forward while standing on a level surface and on a one meter elevated surface. The results showed that in both experiments subjects brought their COP significantly more anterior when the hypothenar eminence of the dominant hand was allowed to lightly slide on the vertical pressure sensitive board even though contact forces on the hypothenar eminence were too small to biomechanically allow support since the forces did not exceed 1 N as measured on the vertical board. It turned out that subjects were able to learn the sliding movement and the number of trials where contact forces were above the threshold were negligibly small.

Forward movement of the COP followed the initial backward displacement and in the touch condition subjects brought their COP further forward on average 5.7 mm on the level ground and 6.1 on the elevated surface. Based on this date we can conclude that the contact at the outreached hand allowed subjects to bring COP further forward. This was observed despite the fact that all of the subjects used a “hip” strategy or anti-phase movement of the pelvis during forward reaching. The results suggest that tactile cues were incorporated into a sensory integration process. It is likely that the addition of touch adds to the building of an internal reference frame as described by Massion et al. [25] but did not influence the movement strategy. The utility of light touch can provide sufficient additional information to the CNS and allows subjects to make extra movement, which he or she would not be willing to do without the utility of light touch. Based on the current results subjects were able to bring the COP further anteriorly as a result of additional sensory flow and further research is warranted to corroborate these results to stability limits expressed as the boundaries of the area over which the centre of gravity may be safely moved [26]. The observed differences between no touch and touch conditions could not be attributed to peripheral mechanisms, namely in young healthy subjects the correlation between the increase of FR in light touch conditions and innervation density of the skin above hypothenar was weak [27]. A possible explanation for the presented results could be found in studies of postural sway with light fingertip contact. A feedback mechanism was proposed by Johanssen et al. [28]. However, in reaching forward a feedback mechanism could not account for the observed response to light touch in the present two experiments. Predictive control processes, on the other hand, could better explain the present results since a feed forward mechanism is responsible for the stabilization of posture during voluntary movements [25] and reaching movement, as performed in the reported experiment, was self-initiated and self-paced movement. Besides additional afferent flow an active attention to light touch was present throughout the experiments. Subjects had to be focused on the sliding with the hypothenar eminence to be able to slide and not to press on the vertical board. We can assume that attention during performance of the movement is another contribution to the processing of the afferent information. This assumption is supported by findings of McNevin and Wolf, [29] who demonstrated that only when subjects were focused on the finger lightly touching was reduction of postural sway possible. In addition, Bolton et al. [30] discovered that cortical activity is associated with active and with task-related touch afferent flow from fingertips and suggested that cortical transmission of sensation from the fingertip is facilitated when the fingertip is in contact with a stable surface.

On the 1 m elevated surface subjects were able to bring their COP further forward with light touch, however the amount of displacement was consistently smaller as compared to the displacement on the level ground. Elevation of 1 m is already considered as a height that imposes postural threat [17], [14] and in no touch conditions a gradual decrease of movement speed of the COP movement has been reported when subjects stood on 1.6 m as compared to a 0.4 m elevated platform [22]. Although the gain of additional forward displacement of COP with light touch was similar in both experiments, when expressed as percentage gain it was grater on elevated surface as compared to excursion on the level ground (13% and 8% respectively) indicating that light touch contributed more to the COP forward movement on the elevation than in the level ground.

During holding of the end position for an agreed 2 seconds two phenomena were observed. First, subjects significantly moved their COP backwards and second sway of COP expressed as path length was observed while holding this position. When comparing the COP forward displacement in touch and no touch conditions we observed in both level and elevated surface that subjects maintained the hold position with light touch at the same distance as was obtained in the maximal forward displacement in the no touch conditions. This result indicates that the subjects were able to use the additional light touch information to maintain their position, which was reached in the no touch condition. We can hypothesise that the reference frame for body position [25] was enhanced by light touch and subjects confidently kept their COP further forward as compared to no contact conditions. These results are in agreement with van Wegen et al. [31] who demonstrated that subjects tend to go away from the maximal end position while attempting to hold it. In the previous studies of COP movements when standing on an elevated surface the shift of COP backwards was reported at 1 m and 1.6 m surface heights [14] and on an even higher - 10.2 m [20]. Besides movements of the COP the reaching movement of the dominant hand was analysed. The results indicate that light touch slightly increases the reaching distance and this increase was statistically significant (p<0.02) on the level as well as on elevated surface. Similar to COP displacement the amount of displacement was consistently smaller on the 1 m elevation as compared to the displacement on the level ground.

On the elevated surface average excursions of COP as well as hand anterior displacement were consistently lesser compared to excursions observed on the level ground. These results are in agreement with previous observations of COP backwards shift during standing on various elevated surfaces [14], [15], [17], [21]. However the addition of light touch allowed subjects to bring their COP and hand further forward in both conditions. On the elevated surface the gain of COP displacement was even five percent larger compared to level ground. This result indicates that subjects were able to partially overcome the decrease in COP excursion imposed by height.

In addition to the analysis of the main variable, the maximal forward displacement of the COP, we analyzed several additional features of COP movement during reaching movements. The first was backward displacement of the COP that preceded the anterior movement of the COP in all experimental conditions. The results of this initial backward displacement of COP was consistent across all experimental conditions and was not influenced neither by afferent flow (touch or no touch conditions) nor by height of the supporting surface. This backward movement represents a backward shift of the pelvis that is associated with forward trunk movement [32]. This activity prior to the reaching movement of the hand accounts for anticipatory postural adjustments (APA) that serve as a stabilizing mechanism for future movements [1] as well as indicating the anti-phase movement strategy (hip strategy) during reaching movement [33]. The amount or magnitude of backward displacement that preceded the forward movement of the COP did not differ between the touch and no touch conditions in our experiments, the APA’s were present in both no touch and touch conditions and the results are in agreement with the prior observations of Massion et al. [34] that the patterns of the anticipatory postural adjustments are fixed and not subject to change, which were observed in experiments in extreme environmental conditions - no or low gravity conditions.

The second, additional variable evaluated was sway of the COP during holding of the final position. Sway of the COP decreased on the level ground as well as on elevated surface. The proposed mechanism for light touch attenuation of postural steadiness was twofold, first information about arm configuration relative to the torso [2] and secondly the increased afferent flow from the whole upper extremity [35] as a consequence of its altered position during touch conditions. In our experiment subjects did not change the position of their hands (inter-segmental position was not altered) between the no touch and touch conditions. Therefore, the increased proprioceptive flow from muscle and joint afferents [35] are not likely to be the explanation for the observed results. In our experimental protocol there was the same position of both hands in touch and no touch conditions thus we believe that the main additional information would be from tactile and not proprioceptive joint afferents. Touch, according to our results functions as “somastetic macula” (Phillips, 1985– cif. Jeka and Lakner [3] not only in the case of opened versus closed eyes (vision or no vision conditions) [2], [3] but also when subjects brought their COP further forward while reaching beyond arm length. These results are in agreement with previous research on the influence of light touch on postural sway [2], [3], [34]. Additionally, besides touch the height of the standing surface in the experiment on the 1 m elevated surface could have influenced postural sway. Based on previous studies, increasing the height of standing (0.4 m, 1 m and 1.6 m) causes the magnitude of sway to decrease [14]. This decrease has been attributed to an increase of stiffness in the ankle region which provides stabilization of the posture. On the other hand, Sitins et al. [21] did not find evidence for increased stiffness around the ankle on a 1 m elevated surface. In our study the observed decrease in sway could be accounted for by both of the previously described mechanisms, the haptic perception and/or increased stiffness in the ankle.

Consecutively repeating the same movement may promote its learning effect [36], therefore we first ensured that subjects performed FR with enough repetitions on the floor in both conditions to minimize or exclude learning effects. Additionally, we repeated the no touch condition and by this confirmed that the differences between no touch and touch conditions were a result of haptic clues and not a result of learning. This enables us a higher degree of confidence that the observed increase of COP and hand forward displacement under light contact conditions is a result of additional sensory flow through the hypothenar region of the dominant hand and not a result of motor learning.

The main limitation of this study is that enrolled subjects were not the same in both experiments but were from the same student population, however the experimental set-up (the position of force platform on a 1 m high box) did not allow us to measure the COP and hand movements in touch and no touch conditions on the level ground and elevated surface in the same experimental session. Not having the possibility to make a within subjects comparison we can only hypothesise that these observed differences are the consequence of elevation of the standing surface.

### Conclusion

In conclusion, the additional haptic information from the hypothenar eminence allowed individuals to bring the COP and hand further forward while attempting to reach forward beyond their arm length while standing on level surface as well as while standing on an elevated surface at a height of 1 m. Light touch on the other hand did not change movement strategies as indicated by initial backward movement of the COP that was consistent through the experimental conditions.
